# Phase-specific functions of macrophages determine injury-mediated corneal hem- and lymphangiogenesis

**DOI:** 10.1038/s41598-018-36526-6

**Published:** 2019-01-22

**Authors:** A. Kiesewetter, C. Cursiefen, S. A. Eming, D. Hos

**Affiliations:** 1 Department of Ophthalmology, University Hospital of Cologne, 50937 Cologne, Germany; 2 Department of Dermatology, University of Cologne, 50937 Cologne, Germany; 3 Center for Molecular Medicine Cologne (CMMC), University of Cologne, 50931 Cologne, Germany; 4 Excellence Cluster: Cellular Stress Responses in Aging-associated Diseases, CECAD, University of Cologne, 50937 Cologne, Germany

## Abstract

Macrophages are critical mediators of injury-associated corneal hemangiogenesis (HA) and lymphangiogenesis (LA). Yet, molecular regulators of the hem- and lymphangiogenic potential of corneal wound macrophages are poorly understood. Using two different mouse models of acute (perforating corneal incision injury) and chronic (corneal suture placement model) corneal injury, here we identified distinct functions of early- versus late-phase corneal wound macrophages in corneal HA and LA. Whereas early-phase wound macrophages are essential for initiation and progression of injury-mediated corneal HA and LA, late-phase wound macrophages control maintenance of established corneal lymphatic vessels, but not blood vessels. Furthermore, our findings reveal that the hem- and lymphangiogenic potential of corneal wound macrophages is controlled by the type of the corneal damage. Whereas perforating corneal incision injury induced primarily wound macrophages with lymphangiogenic potential, corneal suture placement provoked wound macrophages with both hem- and lymphangiogenic potential. Our findings highlight a previously unrecognized injury-context dependent role of early- versus late-phase corneal wound macrophages with potential clinical impact on therapy development for sight-threatening corneal neovascular diseases.

## Introduction

The cornea as the front part and major refractive element of the eye, is physiologically avascular and alymphatic. This corneal avascularity is essential for transparency and is actively maintained by several antiangiogenic mechanisms^1–4^. Corneal wound healing usually proceeds without physiological angiogenesis. However, in case of severe corneal injury the corneal “(lymph)angiogenic privilege” is overpowered by a massive upregulation of pro-angiogenic stimuli leading to an ingrowth of blood vessels (BV) and lymphatic vessels (LV) from the limbal arcade towards the corneal center (corneal neovascularization). Although these neovessels serve to supply cells of the immune system, growth factors and cytokines and in turn mediate their clearance to support corneal wound healing^5^, corneal neovascularization is mostly considered undesirable as ingrowth of BV can interfere with corneal transparency, and lead to lipid deposition and hemorrhage through immature capillaries^3^. LV are clinically invisible and therefore do not noticeably impair corneal transparency but are considered the main risk factor for corneal transplant rejection^6,7^. Corneal LV have additionally been linked to development of ocular surface diseases like dry eye disease and ocular allergy^8,9^. However, we have recently also demonstrated beneficial functions of corneal LV: comparable to LV in cutaneous^10,11^ or intestinal inflammation^12^, corneal LV may support the resolution of persistent inflammation and additionally may be involved in the regulation of corneal edema^5,13^. Therefore, dissecting the cellular and molecular mechanisms that orchestrate the hem- and lymphangiogenic balance in the injured and regenerating cornea is crucial for the development of efficient therapeutic approaches for the treatment and prevention of corneal neovascular diseases, but also to promote corneal repair responses.

Substantial evidence indicates that macrophages are essential mediators of corneal hemangiogenesis (HA) and lymphangiogenesis (LA) after injury^14–16^. It is established that macrophages are able to secrete vascular endothelial growth factor (VEGF)-A, VEGF-C, and VEGF-D that promote vascular endothelial proliferation^14–17^. Our group and others have previously demonstrated that depletion of macrophages decreases angiogenesis in experimental corneal neovascularization^14^ and leads to impaired corneal wound healing in epithelial debridement and corneal transplantation models^18,19^.

Moreover, our group has previously demonstrated that the angiogenic potential of macrophages changes during the progression of skin wound healing: we have shown that especially early stage macrophages recruited in the first hours and days after injury have non-redundant functions for the induction of vascular sprouts and the overall progression of proper skin wound healing, while late stage macrophages rather exert functions on collagen fibril crosslinking and extracellular matrix consolidation^20–22^. Similar dynamics could be involved in corneal angiogenesis^23^. However, it is currently unclear whether macrophages exert different hem-/lymphangiogenic potency during subsequent stages in different settings of corneal damage and how macrophage-mediated angiogenesis supports the corneal repair response. In addition, it is unknown whether macrophages play a role in the maintenance of neovascular structures, which is of particular clinical interest in patients with corneal neovascularization usually presenting with already established corneal neovessels. Thus, in this study we aimed to analyze macrophage dynamics during the corneal inflammatory response after injury and to study the specific function of macrophages during corneal BV and LV initiation, progression, maintenance and regression using phase-restricted depletion of macrophages in subsequent phases after injury. For this purpose, we made use of two corneal injury models in mice: a perforating corneal incision model and the well-established corneal suture model. Both models differ in the kinetics, quality, and quantity of corneal neovascularization. In the incision model a central perforating linear incision disrupting all corneal layers is placed in the corneal center. We have previously shown that this type of injury leads to an isolated growth of LV, but not BV^13^. This lymphangiogenic response is resolved within 4 weeks following injury. Therefore, incision injury is considered as an acute corneal LA model^13^. By contrast, intrastromal suture placement leads to a prolonged chronic injury response. Here three figure-of-eight nylon sutures are placed intrastromally in the peripheral cornea equidistant from the limbus, which leads to epithelial and stromal injury at the site of suture placement^24^. Sutures are left in place for 14 days which leads to a strong inflammatory response as a result of foreign body reaction. Previous work has determined the time course of HA and LA in this model: BV and LV grow into the cornea simultaneously and peak on the day of suture removal. The subsequent vessel regression is faster for LV than for BV, as LV are completely regressed by 6 months, while BV can be detected up to 8 months after suture removal^25^.

Comparing these different injury models allowed to analyze the function of macrophages in HA/LA in acute versus chronic injury responses. Although both models (incision model and suture model) have previously been characterized in their vascular responses after injury^13,25^, a characterization of the interrelationship between macrophage function and vascular growth at different time points after injury is lacking and was the aim of this study.

## Results

### The nature of the corneal damage response determines injury-mediated corneal hem- and lymphangiogenesis

Despite the common association of macrophages with corneal neovascularization, the time-specific function of macrophages in the process of inflammatory corneal HA/LA and wound healing is unknown. To dissect the role of macrophages in inflammatory corneal HA/LA, we made use of two different mouse models of corneal injury: the corneal incision model and corneal suture model. The incision model, which is considered as an acute injury model, leads to transient and isolated LA without concurrent HA^13^, whereas the well-established model of corneal suture placement, which is considered as a chronic injury model, results in parallel growth of corneal BV and LV^25^. In both models the inflammatory macrophage kinetics have not been characterized so far.

**Figure 1:**
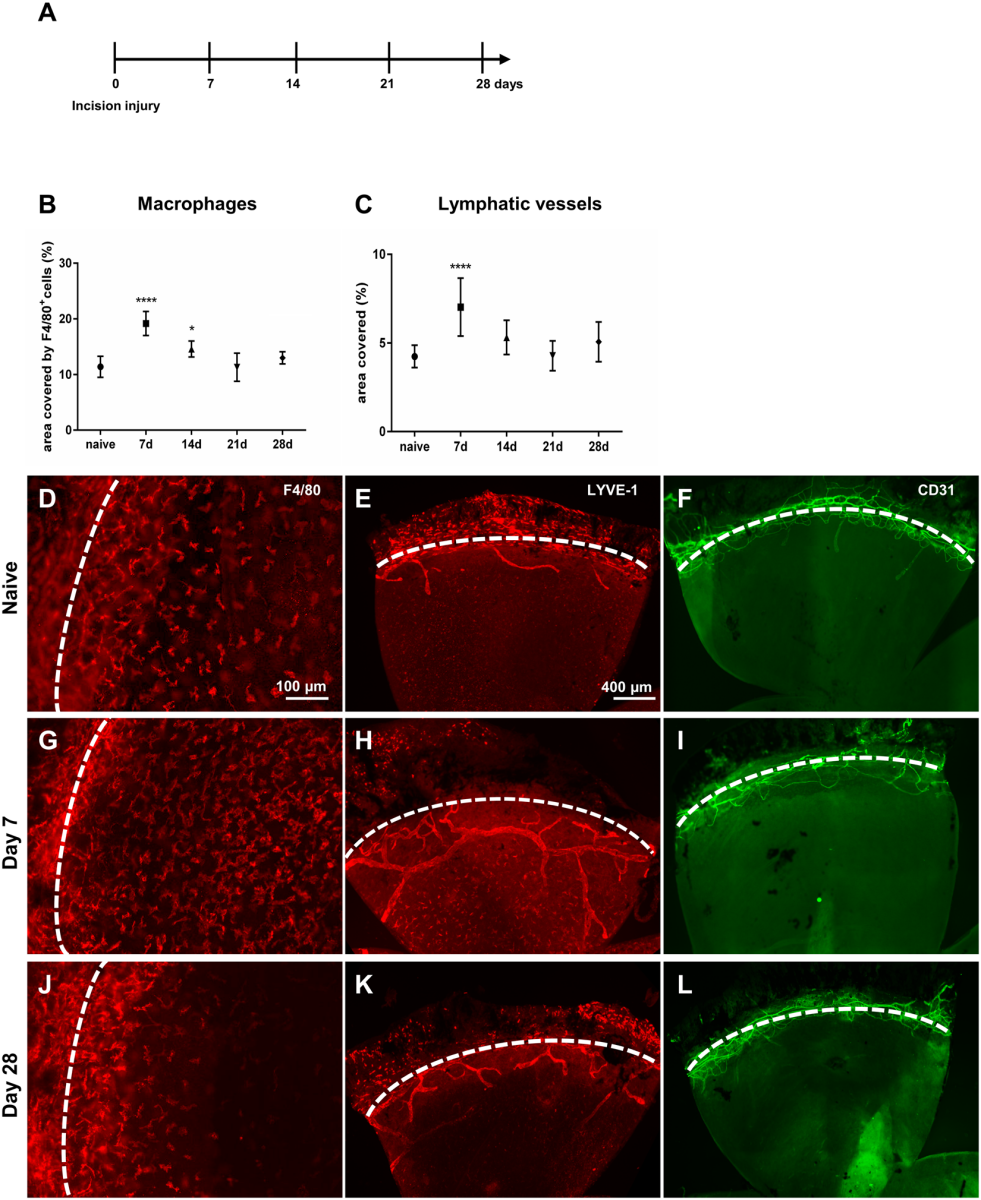
Time course of corneal macrophage infiltrate and lymphangiogenesis after incision injury. (**A**) After incision injury (day 0), the inflammatory macrophage infiltrate, lymphatic vessels (LV) and blood vessels (BV) were monitored at indicated time points. (**B**,**C**) Quantification of total corneal area covered by macrophages and LV at indicated time points. Values were compared to uninjured controls (n = 8–10 per time point). (**D**–**L**) presentative sectors of corneal whole mounts stained for F4/80 (**D**,**G**,**J**), LYVE-1 (**E**,**H**,**K**), and CD31 (**F**,**I**,**L**) at indicated time points after incision injury. Dashed line depicts the corneal limbus. Macrophage numbers in the cornea peak at day 7, and quickly decline. Macrophage numbers are no longer elevated on day 21. LV reach their maximum density on day 7 and start to regress until day 21 when lymphangiogenesis reaches baseline level. Corneal hemangiogenesis is not induced.

To correlate LA and macrophage infiltration after incision injury, we compared the corneal area covered by LV and macrophages at different time points to uninjured controls (Fig. 1). After incision injury, LV sprouted within the first week after injury towards the central incision and reached their maximum on day 7 (7.02% vs. 4.24% uninjured, p < 0.0001). Subsequently, corneal LA quickly declined and LV reached baseline level as soon as 21 days after injury (4.28% vs. 4.24% uninjured) (Fig. 1C). BV sprouting was not induced after incision injury (Fig. 1F,I,L). Similar to LV kinetics, macrophage numbers peaked at day 7 after incision injury (19.17% day 7 vs. 11.38% uninjured, p < 0.0001) and were still elevated on day 14 (14.58% day 14 vs. 11.38% uninjured, p = 0.039). From day 21 onwards macrophage numbers did not differ significantly from control corneas (11.31% day 21 vs. 11.38% uninjured) (Fig. 1B). Thus, our results show that similar to LA, macrophage infiltration is transient after corneal incision injury and rapidly declines with the progression of wound healing, corroborating the acute nature of this model.

**Figure 2:**
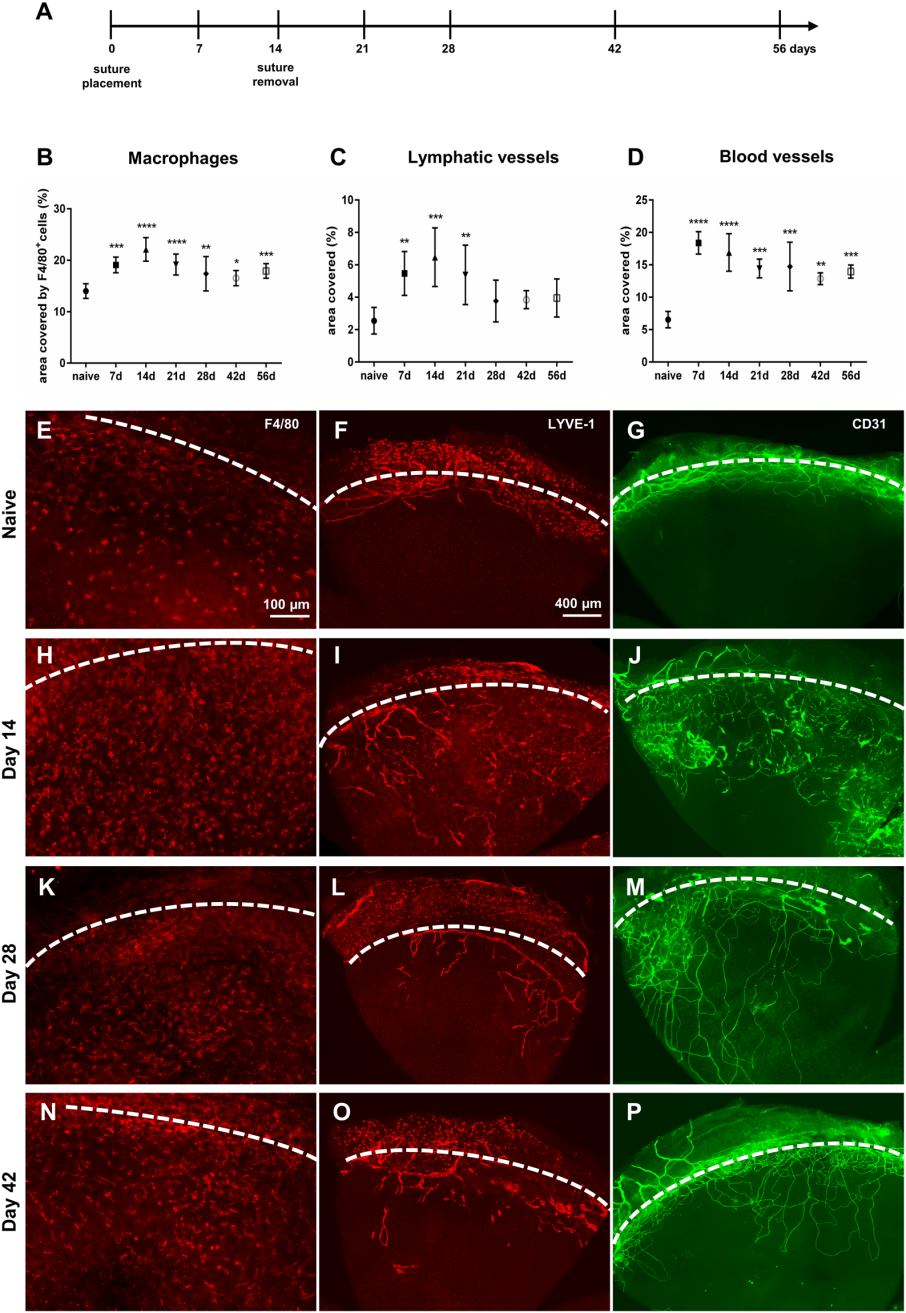
Time course of corneal macrophage infiltrate and hem- and lymphangiogenesis after suture placement. (**A**): After suture placement (day 0), the inflammatory macrophage infiltrate, corneal lymphatic vessels (LV) and blood vessels (BV) were monitored at indicated time points. Sutures were removed on day 14. (**B**–**D)** Quantification of total corneal area covered by macrophages, LV and BV at indicated time points. Values were compared to uninjured controls (n = 8–10 per time point). (**E**–**P**) Representative sectors of corneal whole mounts stained for F4/80 (**E**,**H**,**K**,**N**), LYVE-1 (**F**,**I**,**L**,**O**), and CD31 (**G**,**J**,**M**,**P**) at indicated time points after suture placement. Dashed line depicts the corneal limbus. Macrophage numbers in the cornea peak at day 14, when sutures are removed. The macrophage inflammatory infiltrate in suture injury is long-lasting and still significantly elevated 4 weeks after suture removal. BV and LV reach their maximum density on day 7 and day 14, respectively, and start to regress after subsequent suture removal. BV regression is considerably slower than LV regression.

To study macrophage dynamics in the corneal suture model, we analyzed injured corneas at different time points after suture placement. The corneal area covered by BV, LV and macrophage numbers at each time point were compared to uninjured controls (macrophages: 13.45%, BV: 6.55%, LV: 2.55%) (Fig. 2E–G). Intrastromal suture placement resulted in a strong inflammatory stimulus whereupon macrophages were recruited into the cornea and BV, and LV simultaneously sprouted from the pre-existing limbal vessels towards the corneal center within the first days after injury (Fig. 2). The vascular response appeared faster and stronger than after incision injury, and involved both HA and LA. BV reached their maximum density on day 7 (18.38%) (Fig. 2D), while LV density peaked on day 14 (6.47%) when sutures were removed (Fig. 2C). After suture removal BV and LV regressed only slowly, which is in line with previous reports that have characterized corneal vessel progression and regression after intrastromal suture placement^25^. The amount of BV was still significantly elevated at day 56 after suture placement, which corresponds to six weeks after suture removal (13.96% day 56 vs. 6.55% in uninjured, p = 0.0005). By contrast, LV were mostly regressed at this time point (2.08% day 56 vs. 2.55% uninjured, p = 0.28) and were no longer significantly elevated compared to controls already from day 28 onwards (3.77% day 28 vs. 2.55% uninjured, p = 0.41). Similarly to the quickly developing and relatively long lasting vascular response, macrophage numbers increased quickly after suture placement and peaked on day 14 (22.11% day 14 vs. 13.45% uninjured, p < 0.0001), when sutures were removed (Fig. 2B). After suture removal macrophage numbers did not quickly decline and normalize. A dense macrophage infiltrate was still observed 6 weeks after suture removal (17.93% day 56 vs. 13.45% uninjured, p = 0.0003). Thus, in contrast to the corneal incision model, suture placement not only leads to a long-lasting vascular but also inflammatory response, which can be considered as a model of chronic injury.

In both corneal injury models angiogenesis and macrophage infiltration followed similar kinetics and correlated in time, indicating an interrelation between macrophages and vascular growth. We therefore made use of the different inflammatory and vascular kinetics in both models to analyze the proangiogenic properties of macrophages in steady state and after corneal injury.

### Integrity and maintenance of the resting limbal vasculature is independent from tissue resident corneal macrophages

**Figure 3:**
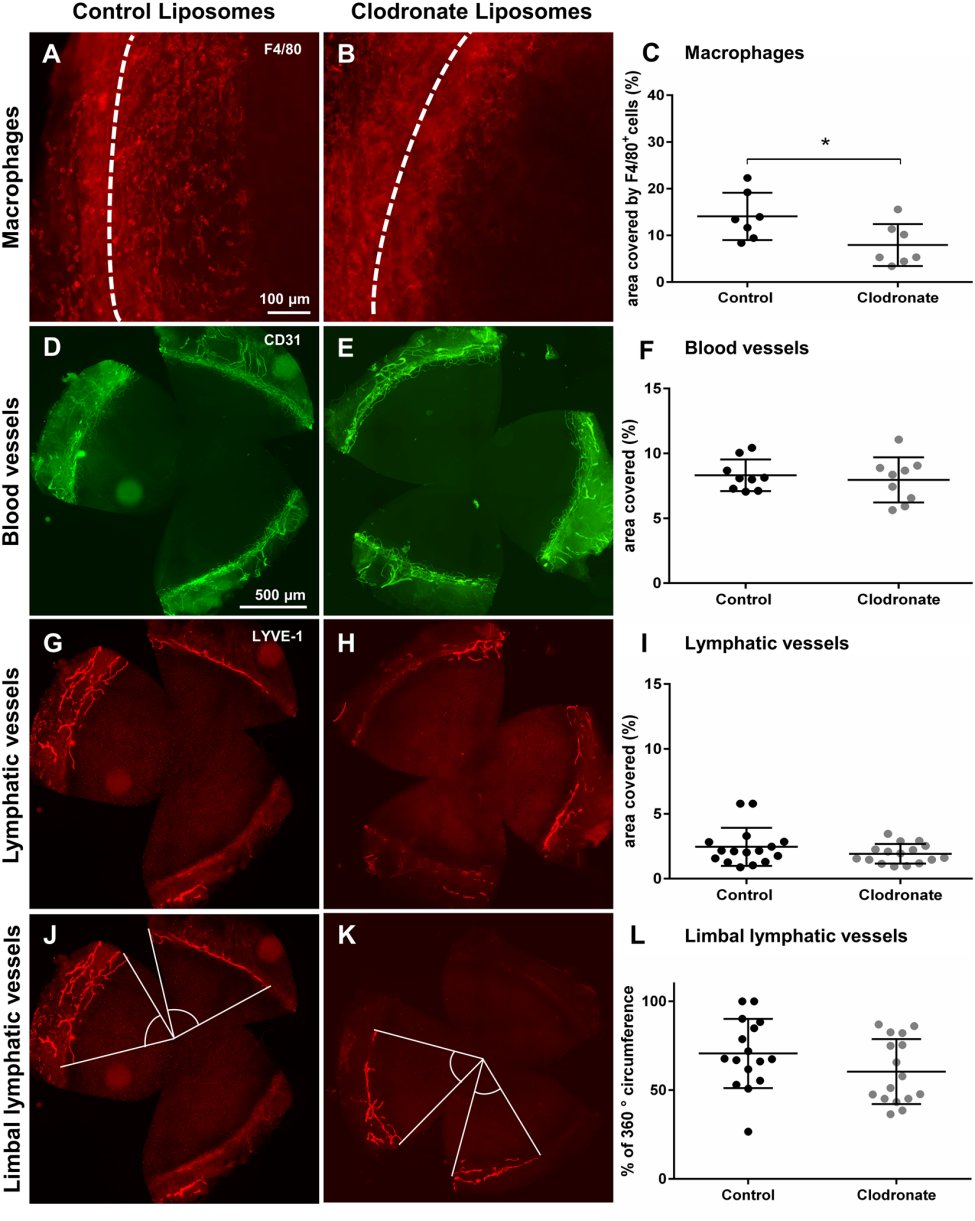
Effect of macrophage depletion on resting corneal limbal vasculature. Representative corneal whole mounts stained for F4/80 (**A**,**B**), CD31 (**D**,**E**) or LYVE-1 (**G**,**H**,**J**,**K**) after macrophage depletion in uninjured corneas. Dashed line depicts the corneal limbus. To analyze the effect of macrophage depletion on the resting limbal blood (BV) and lymphatic vessels (LV), mice received subconjunctival clodronate- or PBS-liposome injections every other day for one week without corneal injury. Macrophage depletion (**A**–**C**) did not reduce the area covered by corneal limbal BV (**D**–**F**) or corneal limbal LV (**G**–**I**) in the resting cornea. **(J**–**L**) The main limbal LV was analyzed by measuring the area of corneal limbus containing a limbal LV, expressed as percentage of total 360 degree circumference. (**C**,**F**,**I**,**L**) Quantification of macrophages and limbal BV and LV (p ≤ 0.05 was considered statistically significant). The limbal BV and LV arcades show no apparent changes after macrophage depletion in uninjured corneas.

In healthy, uninjured corneas, macrophages are frequently localized in the periphery in close proximity to the limbal blood and lymphatic vasculature. Furthermore, limbal LV were previously reported to be sustained by corneal macrophages in C57Bl/6 mice^26^. To analyze whether the steady state maintenance of the limbal blood and lymphatic vasculature in Balb/c mice, which are frequently used in corneal neovascularization models^16,27–29^, also depends on the presence of macrophages, we investigated the impact of macrophage depletion on the resting corneal vasculature in this mouse strain (Fig. 3). Therefore, mice received subconjunctival injections of clodronate liposomes every other day for the duration of one week without corneal injury, which successfully depleted resident corneal macrophages (14.06% control vs. 7.94% depleted, p = 0.03) (Fig. 3A–C). Macrophage depletion did not significantly influence the resting limbal blood (8.32% control vs. 7.96% depleted, p = 0.62) and lymphatic vessel vasculature (2.45% control vs. 1.94% depleted, p = 0.44) (Fig. 3D–I). In addition, we measured the percentage of 360 degree corneal circumference covered by a limbal lymphatic vessel in control versus macrophage depleted corneas as described previously^30^ (Fig. 3J–L). The percentage of corneal circumference covered by a limbal LV was not significantly altered after macrophage depletion, although there was a trend towards a degeneration of the circumferential lymphatic vessel (70.60% control vs. 60.40% depleted, p = 0.14). From these experiments, we conclude that resting limbal BV and LV are not dependent on the presence of macrophages in Balb/c mice under steady state conditions.

### Early stage wound macrophages mediate lymphangiogenesis irrespective of the nature of the corneal damage

**Figure 4:**
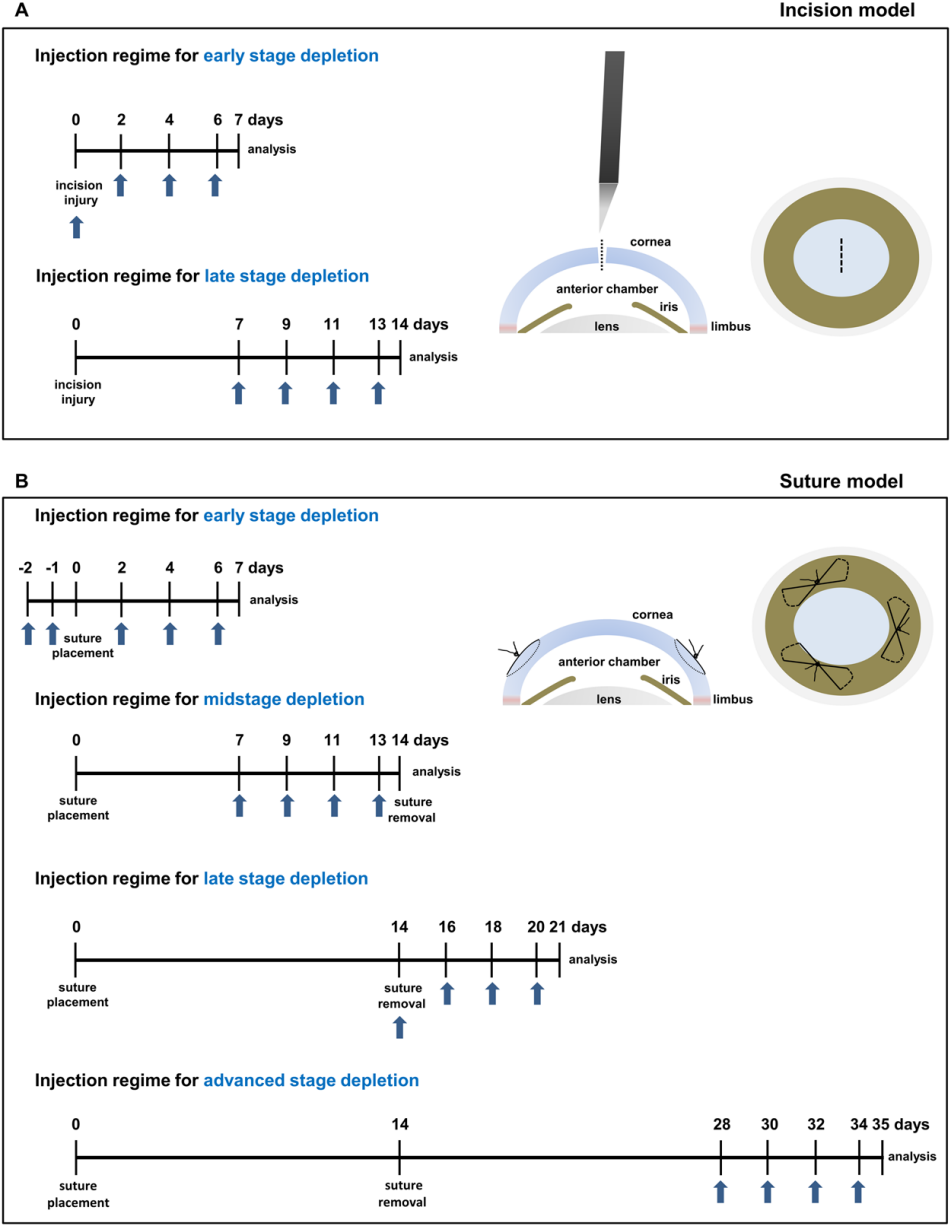
Clodronate liposome injection regimes for stage-specific macrophage depletion in models of corneal injury. Macrophages were depleted by subconjunctival injections of clodronate liposomes at indicated time points (blue arrows) after incision injury (**A**) or suture placement (**B**). Control animals received subconjunctival injections of PBS liposomes.

To dissect the role of macrophages in inflammatory corneal neovascularization after injury, we performed stage-specific depletion of macrophages by subconjunctival injections of clodronate liposomes in different stages of the corneal inflammatory/angiogenic response after both incision and suture injury (depletion schemes are depicted in Fig. 4). After incision injury, macrophages were depleted either in the early phase during the outgrowth of LV (day 0–7) or in the late phase (day 7–14), when most LV are already established (Fig. 4A). After suture placement, macrophages were depleted either in early stage (days 0–7), midstage (days 7–14), late stage (days 14–21), or advanced stage (days 28–35) after suture placement (Fig. 4B) with suture removal performed on day 14. Subconjunctival injections of clodronate liposomes significantly reduced macrophage numbers in the entire cornea at all analyzed time points (representative images of peripheral corneal sections and quantifications of total corneal macrophage numbers are shown in suppl. Figs 1 and 2). Subconjunctivally injected clodronate liposomes also depleted macrophages in the corneal center in uninjured as well as injured corneas, indicating that both corneal resident and recruited macrophages are targeted by subconjunctival clodronate liposome treatment (Suppl. Fig. 3).

**Figure 5:**
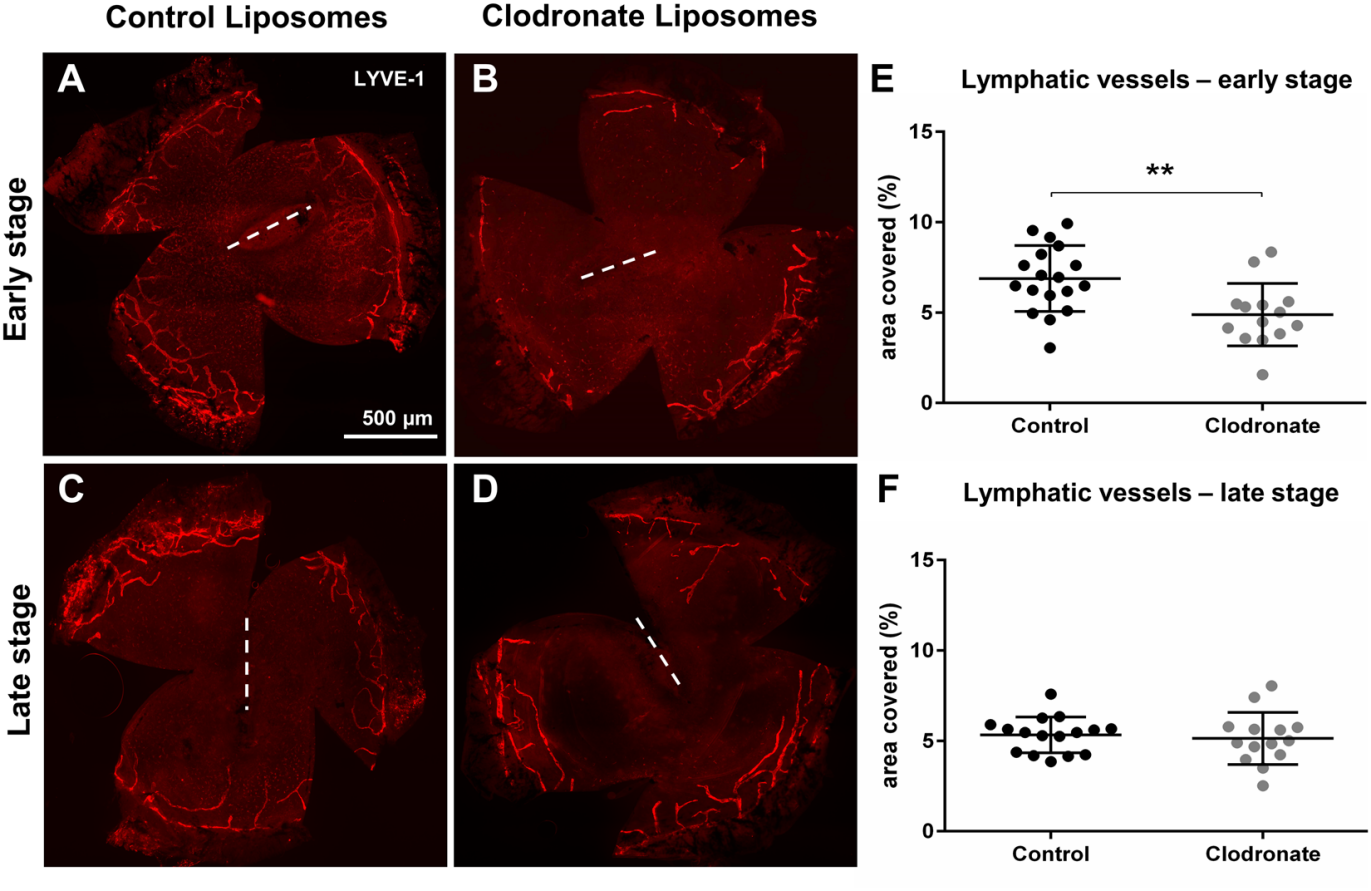
Corneal lymphangiogenesis after stage-specific macrophage depletion after incision injury. Macrophages were depleted during early stage (day 0–7) or late stage (day 7–14) after corneal incision injury, which is characterized by isolated outgrowth of lymphatic vessels (LV). (**A**–**D**) Representative corneal whole mounts stained for LYVE-1 in PBS liposome (control) and clodronate liposome treated mice after early stage (**A**,**B**) or late stage (**C**,**D**) macrophage depletion. Dashed line depicts the area of corneal incision injury. (**E**,**F**) Quantification of LV after early and late stage macrophage depletion (p ≤ 0.05 was considered statistically significant). Depletion of early stage macrophages significantly inhibited outgrowth of LV, while depletion of late stage macrophages did not affect LV.

After incision injury, early stage macrophage depletion strongly reduced inflammatory LA (6.88% control vs 4.87% depleted, p = 0.004) (Fig. 5A,B,E), while depletion of late stage macrophages did not influence LA (5.33% control vs 5.14% depleted, p = 0.67) (Fig. 5C,D,F). Thus, in this setting of acute corneal injury early stage macrophages are essential for the initiation of the vascular response but interestingly only mediate LA, but not HA, which is different to other settings of acute corneal injury such as alkali burn or extensive epithelial debridement where LA is accompanied by HA^31^. Moreover, macrophages seem to be dispensable for late corneal LA and LV maintenance after incision injury.

**Figure 6:**
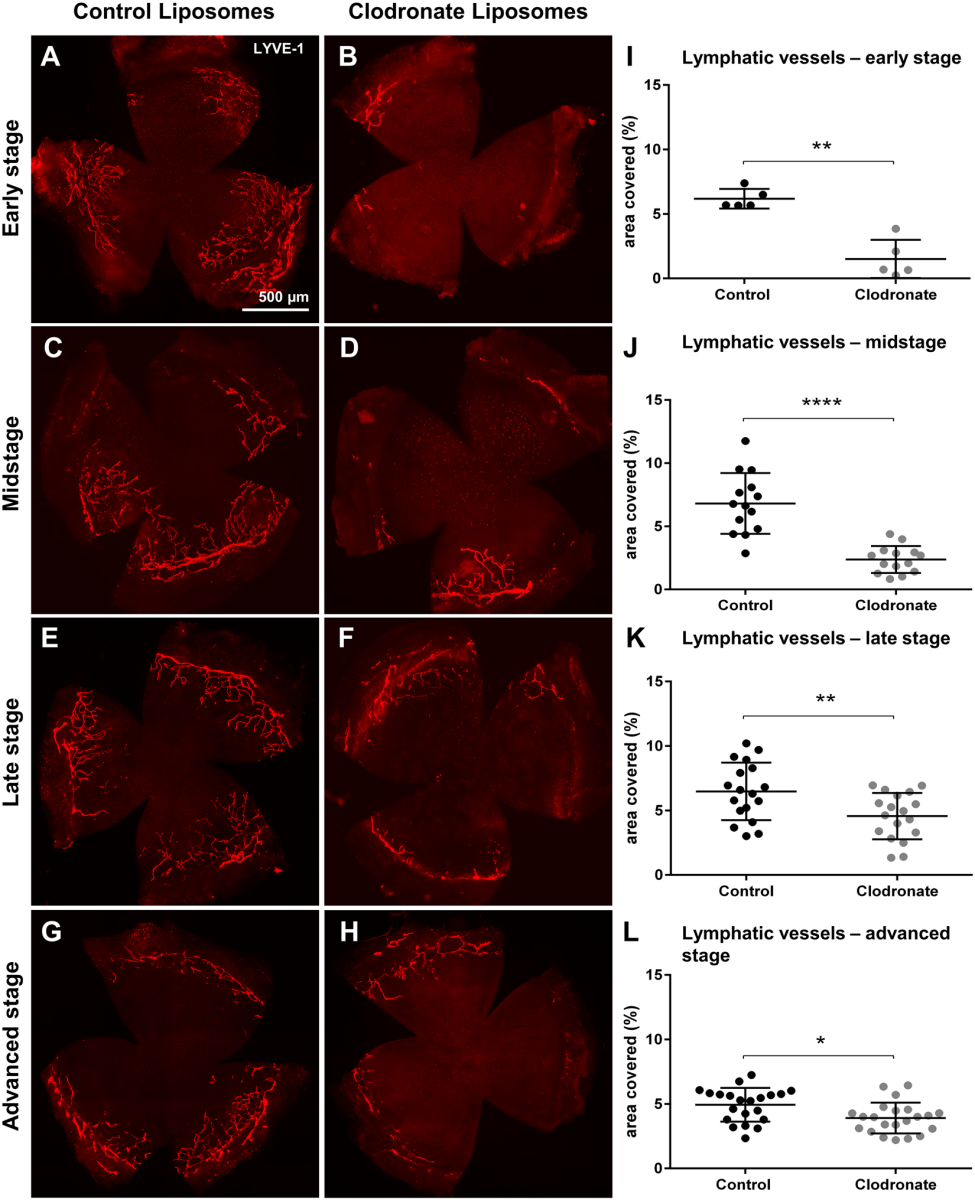
Corneal lymphangiogenesis after stage-specific macrophage depletion after corneal suture placement. Macrophages were depleted by clodronate liposomes either in early stage (days 0–7), midstage (days 7–14), late stage (days 14–21), or advanced stage (days 28–35) after suture placement (suture removal was performed on day 14). (**A**–**H**) Representative corneal whole mounts stained with LYVE-1 in PBS liposome (control) and clodronate liposome treated mice. (**I**–**L**) Quantification of corneal lymphatic vessels (LV) after stage-specific macrophage depletion (p ≤ 0.05 was considered statistically significant). Early and midstage depletion resulted in significant reduction of corneal lymphangiogenesis after suture placement. Late and advanced stage macrophage depletion accelerated LV regression.

Similarly to the incision model, depletion of early stage macrophages after corneal suture placement markedly reduced corneal LA (6.18% control vs. 1.51% depleted, p = 0.008) (Fig. 6A,B,I). Midstage macrophage depletion still resulted in a significant reduction of corneal LA (6.8% control vs. 2.37% depleted, p < 0.0001) (Fig. 6C,D,J). Thus, our results show that early stage macrophages appear to potently induce corneal LA both after acute incision and chronic suture injury.

### Late stage wound macrophages control maintenance of lymphatic vessels in chronically injured cornea

In intrastromal suture-placement both macrophages and neovessels persist long after the inflammatory stimulus is removed. In contrast to acute incision injury angiogenesis is not transient but persistent. To assess whether macrophages in this long-lasting inflammatory infiltrate are involved in maintenance of corneal vessels after removal of the initial stimulus, we performed macrophage depletion in late stages directly after suture removal starting from on day 14 (Fig. 4B, late stage depletion) or starting from day 28 (14 days after suture removal) (Fig. 4B, advanced stage depletion), when macrophage numbers were still significantly elevated. In contrast to our results obtained after acute incision injury where late stage LA was independent of late stage macrophages, late stage LA after chronic suture injury was depended on macrophages: LV regression was accelerated by late stage (6.48% vs. 4.56% p = 0.008) (Fig. 6E,F,K), as well as advanced stage macrophage depletion (4.76% vs. 3.42% p = 0.015) (Fig. 6G,H,L). Thus, late stage macrophages after intrastromal suture placement appear to be involved in LV maintenance, long after the initiating stimulus has been removed.

### Hemangiogenic potential of early stage corneal wound macrophages depends on the type of injury

**Figure 7:**
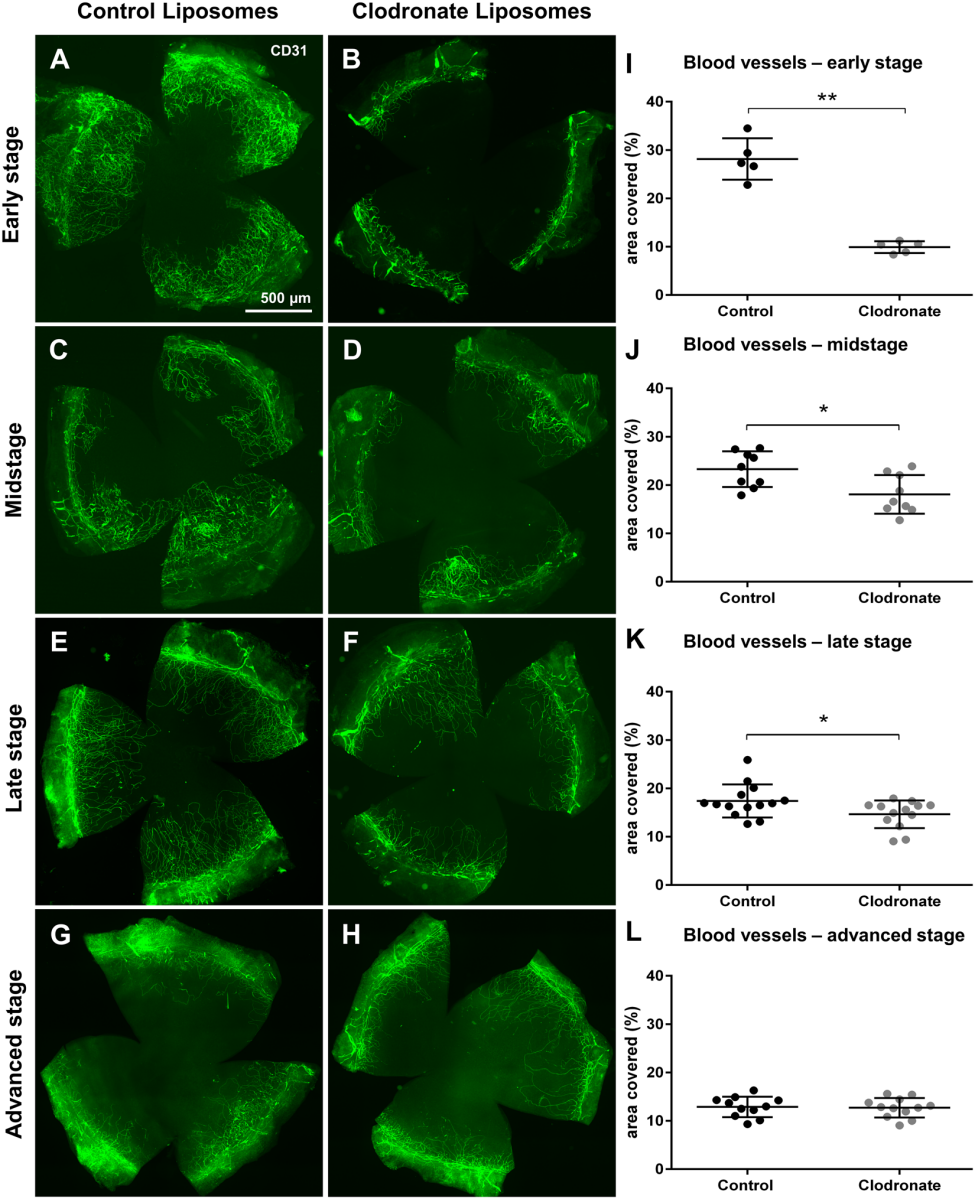
Corneal hemangiogenesis after stage-specific macrophage depletion after corneal suture placement. Macrophages were depleted by clodronate liposomes either in early stage (days 0–7), midstage (days 7–14), late stage (days 14–21) or advanced stage (days 28–35) after suture placement (suture removal was performed on day 14). (**A**–**H**) Representative corneal whole mounts stained with CD31 in PBS liposome (control) and clodronate liposome treated mice. (**I**–**L**) Quantification of corneal blood vessels (BV) after stage-specific macrophage depletion (p ≤ 0.05 was considered statistically significant). Early stage depletion had the most prominent effect on corneal BV after suture placement. The effect of macrophage depletion on corneal hemangiogenesis diminished with time. In advanced stage no accelerated BV regression was observed after macrophage depletion.

In contrast to corneal incision injury (which induces only LV but not BV growth) intrastromal suture placement leads to the simultaneous outgrowth of BV and LV. We therefore analyzed the contribution of macrophages to the initiation, progression, maintenance, and regression of blood vessels after this type of chronic injury. In this setting of corneal injury, ablation of early stage macrophages directly after suture placement strongly reduced the extent of inflammatory HA (28.14% control vs. 9.92% depleted, p = 0.008) (Fig. 7A,B,I), while midstage depletion (1 week after placement) decreased HA to a lesser extent (23.27% control vs. 18.08% depleted, p = 0.01) (Fig. 7C,D,J). Thus, the impact of macrophage depletion on HA in intrastromal suture placement decreased with time. Of note, the overall effect of macrophage depletion on HA was less pronounced than on LA. When assessing the effect of macrophage depletion on the maintenance of BV in late and advanced stage after suture placement, we observed BV to be less dependent on the presence of macrophages than LV. In late stage the effect on BV was subtle but still significant (17.40% vs. 14.63% p = 0.04) (Fig. 7E,F,K), while in advanced stage, regression of BV was not influenced by macrophage depletion (13.20% vs. 12.89%, p = 0.88) (Fig. 7G,H,L), indicating that macrophages are not required for BV maintenance.

### Hem- and lymphangiogenesis after acute/chronic injury depend on macrophage activation

**Figure 8:**
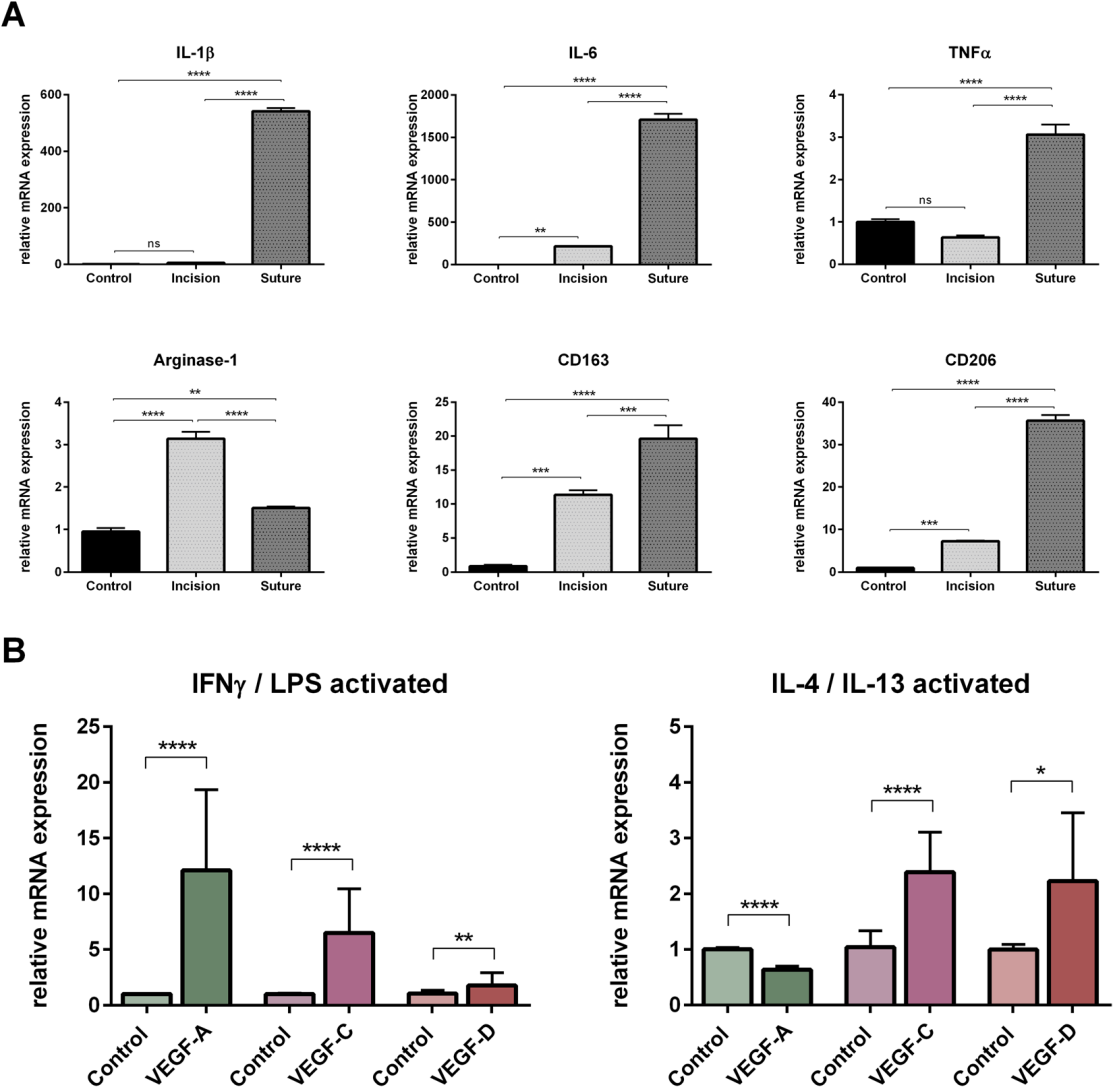
Gene expression analysis of injured corneas *in vivo* and differentially activated macrophages *in vitro*. (**A**) Real-time PCR analysis of corneas on day 7 after incision injury or suture placement. The expression of proinflammatory (type 1 immunity) cytokines IL-1β, IL-6 and TNFα is more pronounced after suture placement than after incision injury. The expression of type 2 cytokine-induced markers Arginase-1, CD163 and CD206 is upregulated after both incision and suture injury, with Arginase-1 being higher expressed in the incision model. One-way analysis of variance (ANOVA). (**B**) Bone marrow-derived IFNγ/LPS- and IL-4/IL-13 cytokine-activated macrophages show different expression of VEGF-A, VEGF-C and VEGF-D. IFNγ/LPS-activated macrophages upregulate VEGF-A, wheras VEGF-A expression is not induced in IL-4/IL-13-activated macrophages. The upregulation of the lymphangiogenic growth factors VEGF-C and VEGF-D is induced in both macrophage subsets. P ≤ 0.05 was considered statistically significant.

In order to better characterize the inflammatory nature and involved macrophage phenotypes in the injury models used in this study, we performed real-time PCR analyses of injured corneas on day 7 after incision and suture injury (Fig. 8A). Expression of the proinflammatory (type 1 immunity) cytokines IL-1β, IL-6 and TNFα was far more pronounced in the suture model (IL-1β: 5.8 fold change after incision vs. 541.5 fold change after suture placement, p < 0.0001; IL-6: 215.6 fold change after incision vs. 1710.0 fold change after suture placement, p < 0.0001; TNFα: 0.6 fold change after incision vs. 3.1 fold change after suture placement, p < 0.0001). Differences in expression of type 2 cytokine-induced markers were less prominent between suture and incision injury. Expression of CD163 (11.4 fold change after incision vs. 19.6 fold change after suture placement, p = 0.0004), and CD206 (7.2 fold change after incision vs. 35.6 fold change after suture placement, p < 0.0001) was induced in both injury models. Similarly, Arginase-1 expression was increased after both injury models, but more pronounced after incision injury than after suture placement (3.1 fold change after incision vs. 1.5 fold change after suture placement, p < 0.0001). These results demonstrate that expression of proinflammatory cytokines is significantly more pronounced after suture injury than after incision injury, indicating an association of type 1 immunity with the suture model. Type 2 activation seems to occur in both injury models. Thus, the suture model is characterized by a more proinflammatory nature, whereas the incision model appears to predominantly involve type 2 immunity.

To further substantiate and link these findings to the expression of proangiogenic factors by macrophages, we isolated bone marrow-derived macrophages and performed real-time PCR after IFNγ/LPS- and IL-4/IL-13 cytokine polarization *in vitro* (Fig. 8B). Expression of the primary hemangiogenic growth factor VEGF-A was upregulated in IFNγ/LPS-activated macrophages (12.1 fold change compared to control, p = 0.0003), but downregulated in IL-4/IL-13-activated macrophages (0.6 fold change compared to control, p < 0.0001). Expression of the lymphangiogenic growth factors VEGF-C and VEGF-D was induced in both IFNγ/LPS-activated macrophages (VEGF-C: 6.5 fold change compared to control, p = 0.0008; VEGF-D: 2.3 fold change compared to control, p = 0.009) and IL-4/IL-13-activated macrophages (VEGF-C: 2.4 fold change compared to control, p < 0.0001; VEGF-D: 2.2 fold change compared to control, p = 0.0124, respectively). This is in line with our *in vivo* findings, where incision injury is more associated with type 2 immunity and the selective induction of LA. In contrast, suture injury seems to be associated with type 1 and type 2 immunity and, resulting in HA as well as LA.

In summary, our results show that early stage corneal wound macrophages initiate LA in both injury models analyzed. While tissue resident macrophages are not involved in LV maintenance under steady state conditions (at least in Balb/c mice), inflammatory macrophages appear to sustain lymphatic neovessel stability after chronic injury, but not after acute injury where LV begin to regress promptly with the progression of wound healing. Furthermore, our results demonstrate that the hemangiogenic potential of early stage corneal wound macrophages strongly depends on the type of injury.

## Discussion

In this study we demonstrated that macrophage infiltration and macrophage-mediated corneal angiogenesis follow different dynamics during diverse settings of corneal injury. We identified distinct functions of early- versus late-phase wound macrophages after corneal injury and showed that the contribution of wound macrophages to the vascular repair response varies during healing progression. While early stage wound macrophages are essential for initiation and progression of both corneal HA and LA, late stage wound macrophages promote HA and LA to a far lesser extent but are involved in the maintenance of established corneal LV, but not BV after injury. These results unravel distinct and phase specific roles for wound macrophages in both vessel progression and maintenance in the injured cornea.

Both injury models analyzed in this study display different vascular responses: acute corneal incision injury leads to transient and isolated outgrowth of LV. By contrast, intrastromal suture placement stimulates both HA and LA, and both vessel types persist for a long time even when the inflammatory stimulus has already been removed. These observations demonstrate that the corneal hem- and lypmphangiogenic response varies depending on the type of injury. Although perforating incision injury results in a disruption of corneal integrity, the wound is quickly closed with epithelium, the anterior chamber is restored within 24 hours, and inflammation is quickly resolved. In this setting of corneal injury, only LV but not BV may be required for the progression of wound healing and the resolution of inflammation and edema^5,13^. By contrast, a chronic foreign body reaction during intrastromal suture placement may require the ingrowth of BV to ensure adequate influx of immune cells, growth factors, and nutrients. Comparing both injury models, it is likely that milder forms of injury only induce the outgrowth of LV to promote wound healing, while concurrent HA is required for proper healing of more severe injuries. Severe injuries might induce HA e.g. to accelerate the healing response or to prevent the transmission of infections to the inner eye, indicating that under these compromised conditions corneal transparency is immolated for the safety of the entire eye. In line with this, isolated LA is also observed in other corneal surface disease models like ocular allergy and dry eye^8,9^, whereas, to our knowledge, no corneal injury model displaying isolated HA exists.

In previous studies macrophages were shown to express the hem- and lymphangiogenic growth factors VEGF-A, VEGF-C and VEGF-D after corneal injury^14,16,17,32^. Although also neutrophils^33,34^ and epithelial cells as well as keratocytes are considered major drivers of corneal angiogenesis due to their secretion of e.g. VEGF, PDGF, FGF and matrix metalloproteinases^35^, macrophages appear to have non-redundant pro-angiogenic functions in vessel progression, as our data demonstrate that early macrophage depletion almost completely abrogated BV and LV sprouting in our injury models.

Phase-specific macrophage depletion after acute incision injury demonstrated that corneal LA in the early phase after injury depends on the presence of prolymphangiogenic macrophages, as early macrophage depletion significantly reduced corneal LA. We have previously shown that corneal incision injury leads to profound corneal edema and that injury-associated corneal LA is involved in the regulation of corneal edema^13^. These data indicate that macrophages are involved in the regulation of corneal volume and fluid homeostasis, arguably via the induction of LV. Indeed, other reports outside the eye have demonstrated similar functions for macrophages^36,37^. Late state depletion of macrophages when LV structures were already established did not influence LA after corneal incision. In this model LV usually quickly regress within 4 weeks after injury without further intervention, as soon as corneal edema is resolved^13^. As LA is transient in the incision injury model, the lymphangiogenic response after injury might not involve a process of vessel maturation. In late stage LV likely have already proceeded to regress and might not depend on macrophage-derived growth factors supporting their maintenance. Additionally, as resolution of edema and LA appear to correlate^13^, corneal edema rather than inflammation might be the driving force for LA in later stages in this injury model, in a process of flow-induced LA^38^. With proceeding resolution of edema, a decline in intravascular flow may therefore initiate LV regression independent of macrophages.

On the contrary, in suture placement a long-lasting inflammatory response appears to drive the angiogenic response and the maintenance of vascular structures. After suture placement, we observed a long-lasting macrophage infiltrate, which persisted even after the sutures were removed. However, from our experiments it is still unclear whether the initial inflammatory macrophage infiltrate is long-lived due to a lack of pro-apoptotic signals or if this observation is a result of ongoing recruitment, or local macrophage proliferation. This dense persisting macrophage infiltrate might account for the induction of HA in addition to LA in this model, assuming that the induction of HA requires higher levels of inflammation and macrophage-derived growth factors when compared to LA. However, the gene expression experiments performed in this study let us conclude that HA might also be mediated by a different subset of macrophages activated after suture placement.

Our analyses revealed that in the suture model, type 1 and type 2 immunity is involved, whereas the predominant immune phenotype in the incision model seems to be type 2. Furthermore, we have shown that VEGF-A is predominantly induced in IFNγ/LPS-activated but not in IL-4/IL-13-activated macrophages, whereas VEGF-C and VEGF-D expression is induced in both macrophage subsets. Therefore, we conclude that incision injury is more associated with type 2 immunity and expression of VEGF-C and VEGF-D (but not VEGF-A), which selectively induces LA. By contrast, suture injury is associated with type 1 and type 2 immunity and expression of VEGF-A, VEGF-C and VEGF-D, resulting in HA as well as LA. Therefore, our findings indicate that the hem- and lymphangiogenic potential of corneal wound macrophages might be controlled by the type of the corneal damage. Certainly the discrimination between different macrophage subsets and activation states in acute versus chronic corneal injury requires further research.

In our experiments of phase-specific macrophage depletion during suture-induced corneal neovascularization, depletion had different outcomes regarding HA and LA. After macrophage depletion LV were considerably reduced at all time points analyzed, while the effect on BV decreased with time. Depletion in early stage following injury resulted in the strongest reduction of corneal HA, whereas depletion at later time points decreased HA to a far lesser extent. In advanced stage, 6 weeks after suture removal, HA was no longer affected by macrophage depletion. Thus, the effect of macrophage depletion in our experiments on HA decreased with time and proceeding vessel maturation. Early stage macrophages infiltrating the cornea directly after injury therefore appear to possess the highest hemangiogenic potential. In line with this, our previous work in skin wound healing demonstrated that early and midstage macrophages are essential for the induction of vascular sprouts and vessel stability, whereas late stage macrophages seem to be dispensable for HA^20^. Also in the cornea, macrophages /macrophage derived factors may be particularly relevant in the sprouting phase of corneal HA, whereas BV maintenance and pruning may proceed independently of macrophages. It is well-established that vessel formation and maintenance are differently regulated^39–41^, which may account for the observed differences in the diverse phases after injury in our study. Otherwise, these results may be explained not by a change in macrophage proangiogenic potential but a decrease in growth factor sensitivity in endothelial cells. During vessel progression in early stage, endothelial cells may rely more on growth factors and may be more sensitive to growth factor deprivation than in later stages. Previous studies have shown that corneal neovessels are quickly covered by pericytes as they mature^42^, which abrogates VEGF responsiveness and promotes the transition into a mature and quiescent state^43–45^. In this regard, numerous studies e.g. in tumor angiogenesis models have shown that lack of pericyte coverage is accompanied by increased sensitivity to VEGF withdrawal^43,46,47^.

In suture-induced neovascularization, corneal LV appeared to be more dependent on the presence of macrophages than BV. Early and midstage depletion strongly reduced LA, indicating that corneal macrophages are essential for LV progression arguably by secretion of lymphangiogenic factors. In late and advanced stage, macrophage depletion still affected LA and led to accelerated regression of LV. The fact that late stage LV are sensitive to macrophage depletion, indicates that LV permanently rely on macrophages/macrophage-derived growth factors, although the specific molecular factors leading to lymphatic endothelial stabilization have not been identified so far. Apart from the secretion of VEGF-C and -D that bind to VEGFR-3 on lymphatic endothelial cells, which promotes cell proliferation, migration and survival^16,48^, macrophages have also been postulated to be directly involved in LV formation. Several groups have observed macrophages integrating into LV like structures and to *de novo* synthesize vessel like structures^15,49–52^, possibly by a process of transdifferentiation into lymphatic endothelial cells^49^. This process of direct integration of macrophages into LV might account for the stronger effect of macrophage depletion on LV than BV. However, the integration of macrophages into LV is still under discussion, as reports using lineage tracing have excluded macrophages as source of lymphatic endothelial progenitors both during embryogenesis and experimentally induced LA e.g. in skin^53^. Nevertheless, macrophages are key regulators of LA, although the precise mechanisms are not completely understood. Moreover, apart from their influence on LV sprouting after injury, macrophages in the cornea were previously reported to sustain the limbal lymphatic vasculature in the resting cornea, at least in C57Bl/6 mice^26^. In our current experiments we were not able to detect significant changes in the limbal blood and lymphatic vasculature following macrophage depletion in Balb/c mice, which may account for strain specific differences in corneal macrophages or LV^54,55^. Indeed, it has been shown that corneas of Balb/c mice contain fewer numbers of resident macrophages and possess discontinuous limbal lymphatic vessels^26,54^, which might be responsible for the observed differences in vessel maintenance under steady state conditions.

In summary, our results imply that macrophage-targeted treatment strategies may only be sufficient to attenuate BV progression in the cornea but may not provide treatment options to accelerate BV regression, the latter being of high clinical relevance. By contrast, targeting macrophages to facilitate a faster regression of LV might be of clinical use in treating corneal surface diseases that have been shown to be mediated by persistent corneal LV such as dry eye disease and ocular allergy^8,9^. Additionally, these strategies could be implemented to reduce graft rejection after corneal transplantation, as we and others have previously shown that especially LV mediate immune rejection episodes after corneal grafting^1,7^.

## Methods

### Animals

All animal experiments were approved by the local animal care committee (LANUV, Approval No. 84–02.04.2015.A487) and were performed in accordance with the Association for Research in Vision and Ophthalmology Statement for the Use of Animals in Ophthalmic and Vision Research. Mice were kept under standard pathogen-free conditions in a 12 h light-dark cycle. Prior to suture placement, suture removal, corneal incision and subconjunctival injections, mice received injection anesthesia consisting of ketamine (100 mg/kg) and xylazine (20 mg/kg). Additionally, oxybuprocaine hydrochloride eye drops (Conjuncain edo, Bausch & Lomb, Berlin, Germany) were applied as local anesthetic.

### Mouse model of corneal incision injury

Corneal perforating incision injury was performed in the right cornea of 6–8 week old female C57Bl/6 N mice (obtained from Charles River Laboratories, Sulzfeld, Germany), as previously described^13,56^. Mice received atropine sulfate eye drops (Atropin POS 1%, Ursapharm GmbH, Saarbruecken, Germany) 20 minutes prior to surgery to avoid iris incarceration. To generate a perforating corneal incision with 1 mm length, the corneal center was marked with a 1.0 mm trephine. The cornea was perforated with a 30-gauge needle and a sagittal linear incision was inflicted using surgical micro scissors. After incision injury, mice received ofloxacin eye drops (Ofloxacin-ratiopharm, ratiopharm GmbH, Ulm, Germany) three times daily during the first three days.

### Mouse model of suture-induced corneal neovascularization

The mouse model of suture-induced corneal neovascularization was performed as previously described^14,57^. Briefly, three figure-of-eight 11-0 nylon sutures (Serag Wiesner, Naila, Germany) were placed intrastromally in the cornea of the right eye of 6–8 week old female Balb/c mice (obtained from Charles River Laboratories, Sulzfeld, Germany). Each figure of eight-suture was placed with two stromal incursions extending over 120 degrees of the corneal circumference. Sutures were left in place for 14 days, if not indicated otherwise.

### Experimental macrophage depletion

For macrophage depletion mice received subconjunctival injections of 10 µl mannosylated clodronate liposomes (Encapsula NanoSciences LLC, Brentwood, USA) every other day for the duration of one week. Control mice received equal amounts of mannosylated PBS-Liposomes. To deplete corneal macrophages at different time points after suture placement and incision injury, clodronate liposomes were administered in a time restricted manner using different injection regimens (Fig. 4). At the end of each experiment, corneas were excised and stained for vascular markers to quantify the extent of corneal HA and LA.

### Immunohistochemistry of corneal whole mounts

Mice were sacrificed at the indicated time points, and corneas were excised, rinsed in PBS, and incubated in 20 mM EDTA in PBS for 30 min at 37 °C for removal of the corneal epithelium. Subsequently, corneas were washed, fixed in ethanol, and blocked in 2% BSA. For the analysis of corneal macrophages, BV, and LV, corneas were stained with FITC-conjugated anti-CD31 (BD Biosciences, Heidelberg, Germany) and anti-LYVE-1 (lymphatic vessel endothelial hyaluronan receptor 1) (Acris Antibodies GmbH, Herford, Germany), or anti-F4/80 (Invitrogen, Eugene, USA) overnight. To detect LYVE-1 or F4/80 corneas were washed and incubated with Cy3 goat-anti rabbit (Dianova, Hamburg, Germany) or Alexa fluor 555 goat anti-rat secondary antibody (Invitrogen, Eugene, USA) for 45 min. After final washing, corneas were mounted on microscope slides with fluorescence mounting medium (Dako, Glostrup, Denmark).

### Evaluation of corneal hem- and lymphangiogenesis

Double-stained corneal whole-mounts were analyzed in 100-fold magnification with a fluorescence microscope (Olympus BX53, Olympus Optical Co, Hamburg, Germany). Semi-automatic morphometric image analysis was performed using Olympus CellF software (Olympus Soft Imaging Solutions GmbH, Muenster, Germany) as described previously^57^. Briefly, for the detection of bright vessels on dark background, gray-scale pictures of fluorescence stained corneal whole-mounts were modified using several software-based filters to sharpen images and increase contrast. After manually setting a threshold for detection, bright corneal vessels were detected on black background in an automatic manner. Results are expressed as the percentage of corneal area covered by BV or LV. The total corneal area was defined using the outermost corneal limbus vessel as outer limiting border (Suppl. Figs 4 and 5). Macrophage numbers were expressed as percentage of corneal area covered by F4/80 positive cells. For analysis of the limbal circumferential vasculature, which is discontinuous in Balb/c mice^58^, angles of the cornea containing a main limbal LV were measured and expressed as percentage of the total 360° circumference, as described previously^30,50^.

### RNA isolation and real-time PCR analysis

On day 7 after incision or suture injury, central corneas (n = 3 per group) sparing the limbal area were excised and placed into RNA later solution (Qiagen, Hilden, Germany) until further processing. Tissue disruption was performed using Precellys 24 tissue homogenizer (Bertin Instruments, Montigny-le-Bretonneux, France). RNeasy Micro Kit (Qiagen, Hilden, Germany) was used for total RNA isolation. Complementary DNA (cDNA) synthesis was performed in random hexamer primed reverse transcriptase reactions (SuperScript III; Invitrogen, Carlsbad, CA). Primers for real-time PCR (Eurofins Genomics, Ebersberg, Germany) were designed using Primer3 software and BLAST (Basic Local Alignment Search Tool, NCBI). Primer sequences are provided in suppl. table 1. PCR reactions contained 0,5 µM of each Primer, 10 ng cDNA and SYBR green master mix (SsoFast EvaGreen Supermix; Bio-Rad, Hercules, CA) in a total volume of 25 µl. Real-time PCR analyses were performed in triplicates under the following conditions: 95 °C for 2 minutes, 40 cycles at 95 °C for 5 seconds and 60 °C for 15 seconds (PCR Cycler CFX96 Bio-Rad, Hercules, USA). Hypoxanthine guanine phosphoribosyl transferase (HPRT) was used as reference gene. Gene expression was normalized to expression levels in uninjured corneas using the ∆∆CT method.

### Analysis of bone marrow-derived macrophages

Bone marrow-derived macrophages were isolated from female C57Bl/6 N mice (n = 3) according to standard protocols. Cells were cultured for 7 days in RPMI medium (Life technologies, Eugene, USA) containing 10% FBS (Life technologies, Eugene, USA) in the presence of 20 ng/ml M-CSF (Miltenyi Biotec, Bergisch Gladbach, Germany). Afterwards, macrophages were detached from culture dishes using accutase solution (PromoCell, Heidelberg, Germany) and 1 × 10^6^ cells per group were seeded in 6-well plates and allowed to re-adhere overnight. The following day macrophages were stimulated with 100 ng/ml IFNγ and 10 ng/ml LPS (E.coli LPS, Sigma Aldrich, St. Louis, USA) or IL-4 and IL-13 (100 ng/ml each). Cytokines were obtained from PeproTech (Rocky Hill, USA). Control cells were left unstimulated. Total RNA from macrophages was isolated 24 h later using QIAshredder columns (Qiagen, Hilden, Germany) for cell disruption and the Qiagen RNeasy Micro Kit (Qiagen, Hilden, Germany). cDNA synthesis and real-time PCR were performed as described above.

### Statistical analyses

Statistical analyses were performed using GraphPad Prism version 6.07 for Windows (GraphPad Software Inc., La Jolla, California, USA). Significance was analyzed using Student’s t-test for parametric or Mann-Whitney-U test for non-parametric data sets. One-way analysis of variance (ANOVA) was used to compare BV, LV and macrophage numbers at different time points to uninjured controls and to compare mRNA expression levels in corneal tissue. A value of p ≤ 0.05 was considered statistically significant. All data are presented as single or grouped values with mean and standard-deviation (SD).

## Electronic supplementary material

Supplementary Dataset 1

## Data Availability

The datasets generated during and analyzed in this study are available from the corresponding author on reasonable request.
