# Supplementary material for: Phase-specific functions of macrophages determine injury-mediated corneal hem- and lymphangiogenesis
Source: Sci Rep. 2019 Jan 22;9:308. doi: 10.1038/s41598-018-36526-6 (PMC6343005; doi:10.1038/s41598-018-36526-6)
Supplement: Supplementary file 1 — Supplementary Dataset 1 [file 41598_2018_36526_MOESM1_ESM.docx]

**Phase-specific functions of macrophages determine injury-mediated corneal hem- and lymphangiogenesis**

Kiesewetter A.^1, 2^, Cursiefen C.^1, 3^, Eming S. A.^2, 3, 4*^, Hos D.^1, 3*^

^1^ Department of Ophthalmology, University Hospital of Cologne, 50937 Cologne, Germany.

^2^ Department of Dermatology, University of Cologne, 50937 Cologne, Germany.

^3^ Center for Molecular Medicine Cologne (CMMC), University of Cologne, 50931 Cologne, Germany.

^4^ Excellence Cluster: Cellular Stress Responses in Aging-associated Diseases, CECAD, University of Cologne, 50937 Cologne, Germany.

*Corresponding authors:

Deniz Hos, MD, PhD, Department of Ophthalmology, University of Cologne, Kerpener Strasse 62, 50924 Cologne, Germany; phone: +49 221 478 -4300; fax: -5094; deniz.hos@uk-koeln.de; www.augenklinik.uk-koeln.dewww.augenklinik.uk-koeln.de

Sabine A. Eming, MD, Department of Dermatology, University of Cologne, Kerpener Strasse 62, 50937 Cologne, Germany; phone +49 221 478-3196; fax -1424540; sabine.eming@uni-koeln.de

**Supplemental figures:**

**
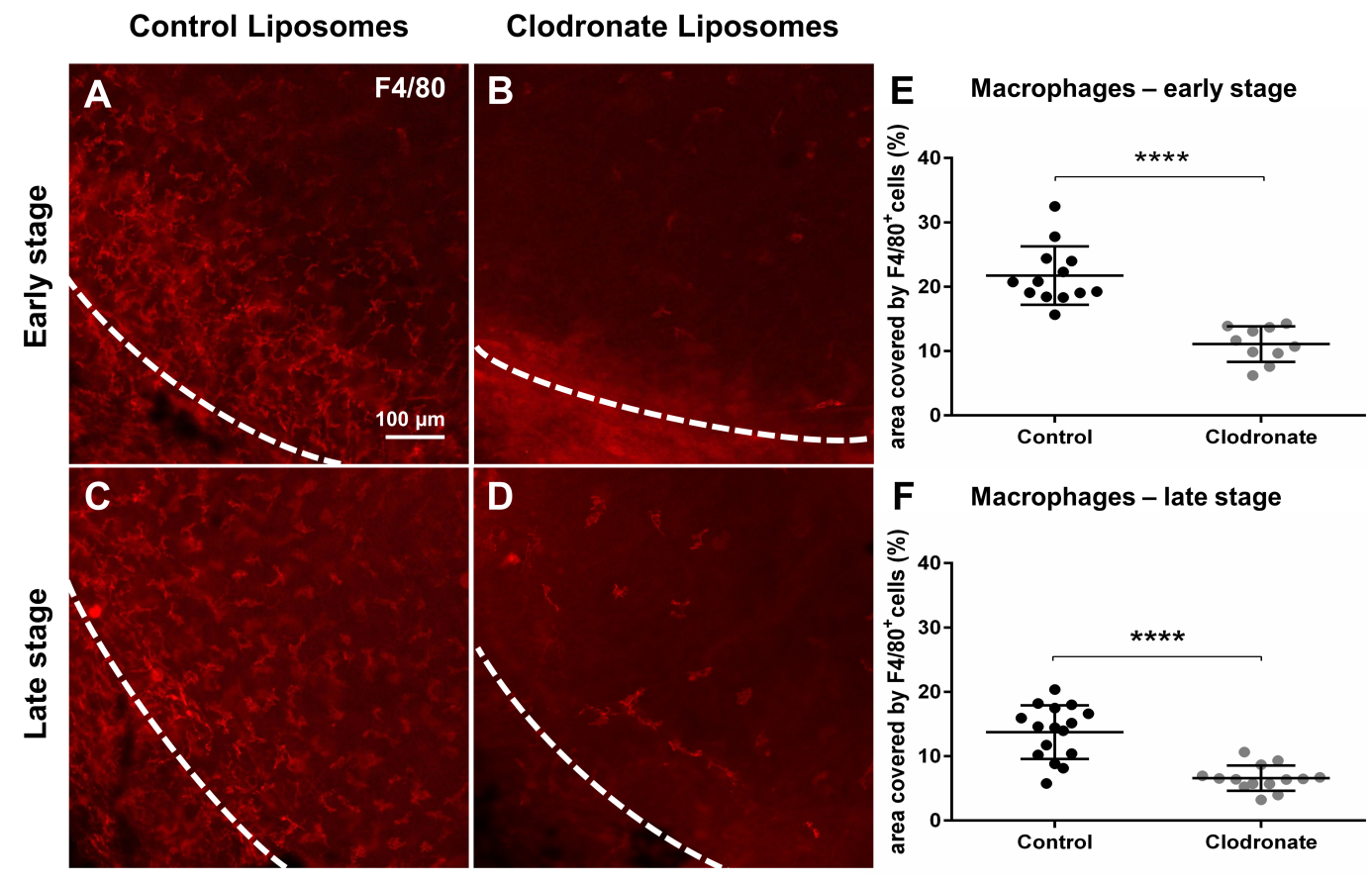
**

**Supplemental Figure 1. Efficiency of stage specific-macrophage depletion after incision injury.** Macrophages were depleted by subconjunctival injections of clodronate liposomes either in early stage (days 0-7), or late stage (days 7-14) after corneal incision injury. Afterwards, corneas were excised and stained for F4/80 (**A-D**) and the percentage of corneal area covered by macrophages was quantified (**E,F**). Application of clodronate liposomes effectively depleted corneal macrophages at both time points. **E**: Quantification of macrophages after early stage depletion, p= 0.0036. **F:**Quantification of macrophages after late stage depletion, p< 0.0001. Dashed line depicts the corneal limbus.

**
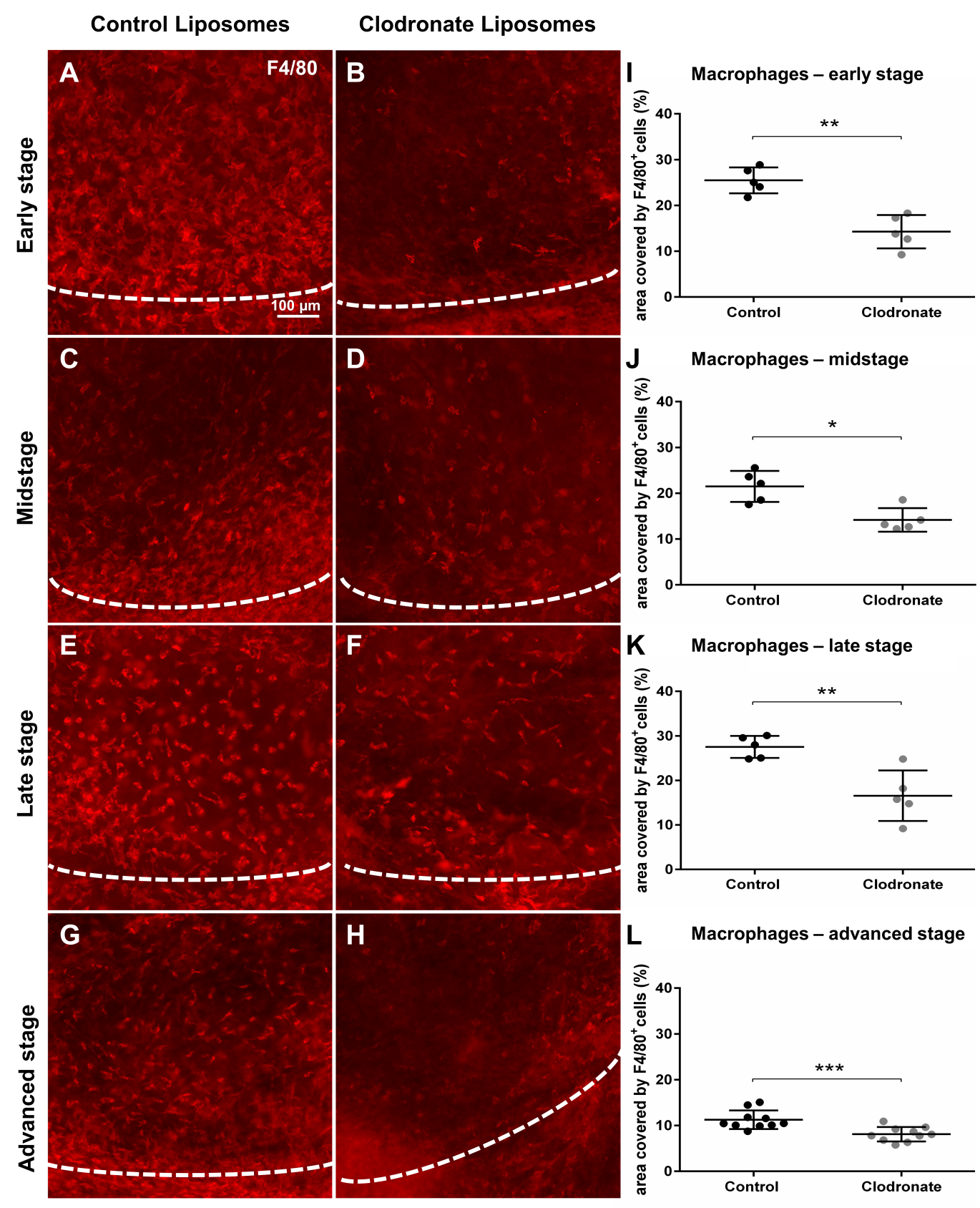
**

**Supplemental Figure 2. Efficiency of stage specific-macrophage depletion after suture placement.** Macrophages were depleted by subconjunctival injections of clodronate liposomes either in early stage (days 0-7), midstage (days 7-14), late stage (days 14-21), or advanced stage (days 28-35) after suture placement. Suture removal was performed on day 14. Afterwards, corneas were excised and stained for F4/80 (**A-H**) and the percentage of corneal area covered by macrophages was quantified (**I-L**). Application of clodronate liposomes effectively depleted corneal macrophages at all time points. **I**: Quantification of macrophages after early stage depletion, p= 0.008. **J:** Quantification of macrophages after midstage depletion, p= 0.03. **K**: Quantification of macrophages after late stage depletion, p= 0.008. **L**: Quantification of macrophages after advanced stage depletion, p= 0.0007. Dashed line depicts the corneal limbus.


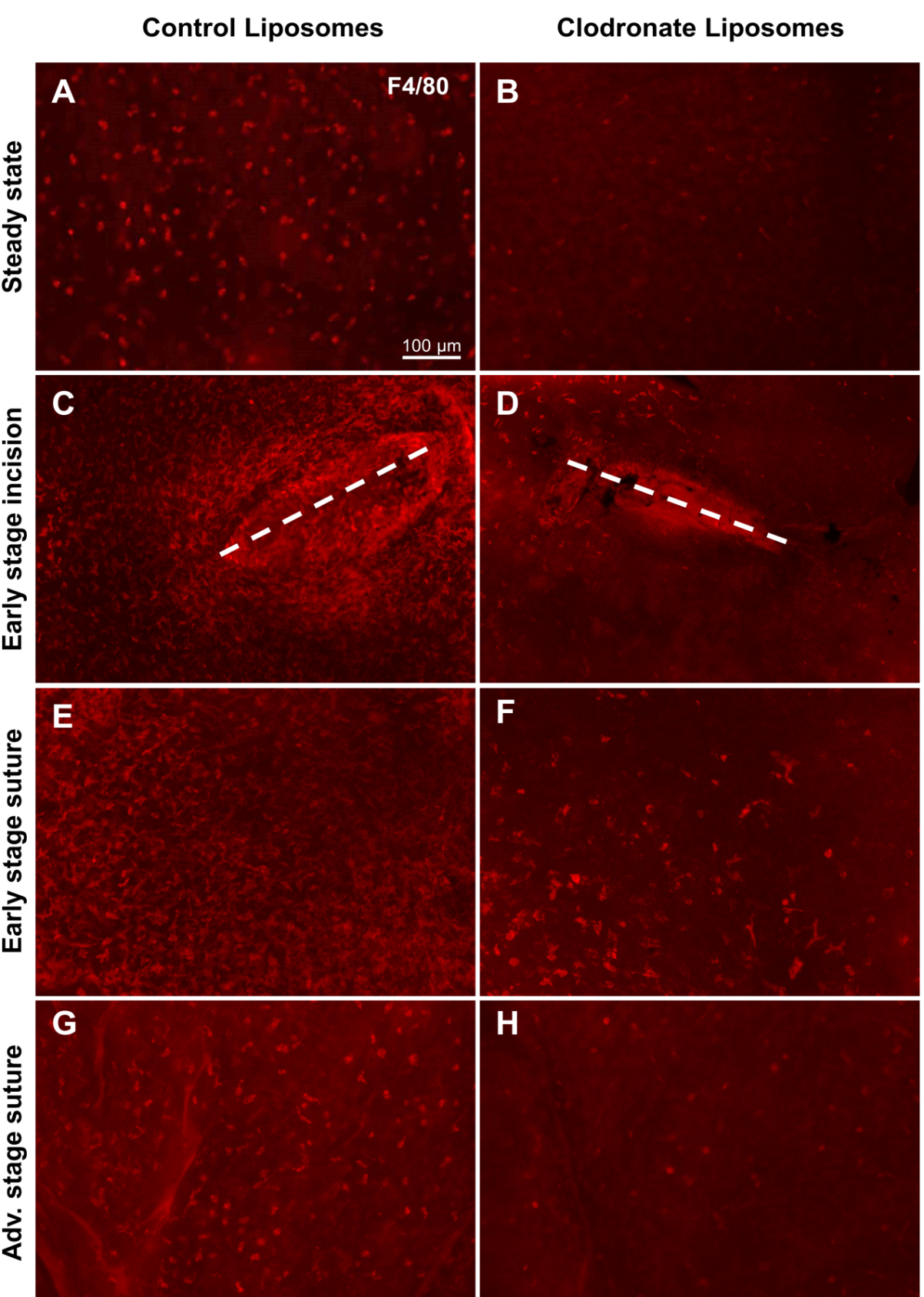


**Supplemental Figure 3. Efficiency of macrophage depletion in the central cornea.** Representative sections of central corneal whole mounts stained for F4/80 in clodronate liposome treated and PBS liposome treated control mice. Repeated subconjunctival injections of clodronate liposomes successfully depleted macrophages in the corneal center in uninjured corneas (**A, B**), in early stage (days 0-7) after incision injury (**C,D**), in early stage after suture injury **(E,F**), and advanced stage (days 21-28) after suture injury (**G, H**). Dashed line in C and D depicts the corneal incision.

**
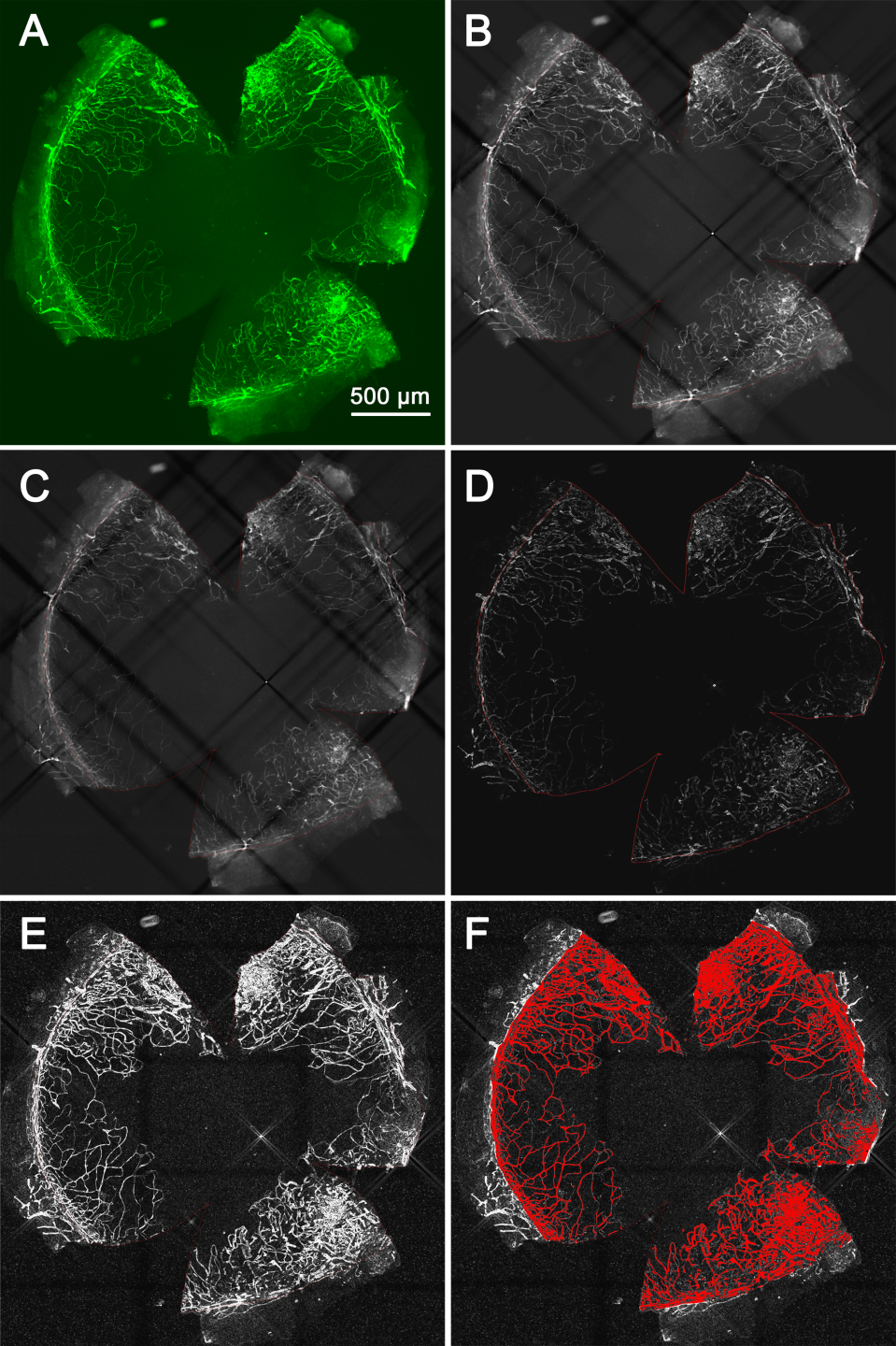
**

**Supplemental Figure 4. Quantification of corneal blood vessels.** For quantification of corneal blood vessels, grey scale pictures of CD31 stained corneal whole mounts (**A**) were modified using several software based filters to increase contrast and sharpness of the images (**B-E**). Afterwards, corneal vessels were detected in a semi-automatic manner, using the outermost limbal blood vessel as limiting border (**E**). Values were expressed as percentage of total corneal area covered by vessels.

**
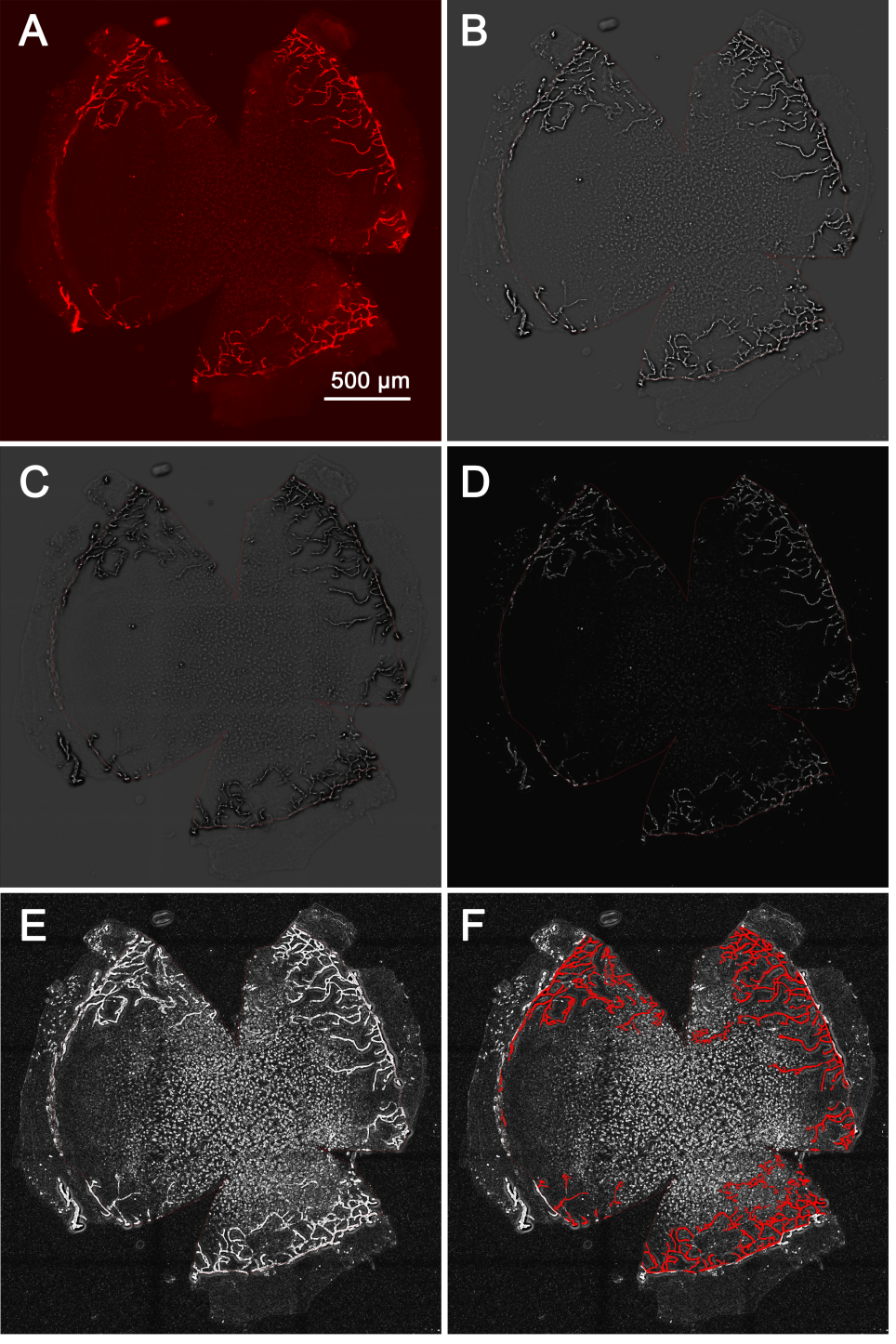
**

**Supplemental Figure 5. Quantification of corneal lymphatic vessels.** For quantification of corneal lymphatic vessels, grey scale pictures of LYVE-1 stained corneal whole mounts (**A**) were modified using several software based filters to increase contrast and sharpness of the images (**B-E**). Afterwards, corneal vessels were detected in a semi-automatic manner, using the outermost limbal lymphatic vessel as limiting border (**E**). Values were expressed as percentage of total corneal area covered by vessels.

| **Primer** | **Sequence forward (5’-3’)** | **Sequence reverse (5’-3’)** |
| --- | --- | --- |
| Arginase-1 | GCAGAGGTCCAGAAGAATGG | GTGAGCATCCACCCAAATG |
| CD163 | GGCACTCTTGGTTTGTGGAG | GCCTTTGAATCCATCTCTTGG |
| CD206 | TGCCGACATGCCAGGACGAAA | GTGGGCTCTGGTGGGCGAGT |
| IL-1β | GTCCTGTGTAATGAAAGACGGC | CTGCTTGTGAGGTGCTGATGTA |
| IL-6 | ACACATGTTCTCTGGGAAATC | AAGTGCATCATCGTTGTTCATACA |
| TNFα | AGGACTCAAATGGGCTTTCC | CAGAGGCAACCTGACCACTC |
| VEGF-A | CATGGATGTCTACCAGCGAAG | CATGGTGATGTTGCTCTCTGAC |
| VEGF-C | AGAACGTGTCCAAGAAATCAGC | ATGTGGCCTTTTCCAATACG |
| VEGF-D | ATGGCGGCTAGGTGATTCC | CCCTTCCTTTCTGAGTGCTG |

**Supplemental Table 1. Primer sequences used for real-time PCR**
